# Daratumumab prevents programmed death ligand‐1 expression on antigen‐presenting cells in de novo multiple myeloma

**DOI:** 10.1002/cam4.2827

**Published:** 2020-01-28

**Authors:** Nicolas Stocker, Béatrice Gaugler, Laure Ricard, Frédéric de Vassoigne, Zora Marjanovic, Mohamad Mohty, Florent Malard

**Affiliations:** ^1^ INSERM Centre de Recherche Saint‐Antoine (CRSA) Sorbonne Université Paris France; ^2^ AP‐HP Hôpital Saint‐Antoine Service d’Hématologie Clinique Paris France

**Keywords:** antigen‐presenting cells, daratumumab, multiple myeloma

## Abstract

**Background:**

Daratumumab (Dara), an anti‐CD38 monoclonal antibody, has an immunologic mechanism of action through targeting of CD38 expressing immune cells in patients with multiple myeloma (MM). Furthermore, it was recently shown that CD38 upregulation in tumors, is a major mechanism of acquired resistance to antiprogrammed cell death 1 (PD‐1)/programmed cell death ligand 1 (PD‐L1). Therefore, we decided to evaluate the immunomodulatory effects of CD38 blockade by Dara on the PD‐L1 expressing immune cells.

**Methods:**

We analyzed CD38 and PD‐L1 expression on immune cells at different time points in 18 newly diagnosed MM receiving bortezomib, lenalidomide and dexamethasone, with or without Dara.

**Results:**

We first confirmed that CD38 is widely expressed on immune cells, with the strongest expression on plasmacytoid dendritic cells (pDC). Furthermore, Dara induces a strong depletion of pDC in addition to the well‐known rapid depletion of natural killer cells. Finally, we found that PD‐L1 expression on antigen‐presenting cells (APC) increases with MM treatment in patients that did not received Dara, while addition of Dara prevents this increase.

**Conclusion:**

Overall, our results suggest new mechanisms of action of Dara through depletion of pDC and prevention of PD‐L1 upregulation expression on APC. Our finding provides new evidences for development of therapeutic strategies targeting both CD38 and PD‐L1/PD‐1 pathway in patients with MM.

## Graphical abstract

Daratumumab have immunomodulatory effects on CD38‐expressing immune cells. Daratumumab prevents PDL1 expression on CD38‐expressing antigen‐presenting cells.

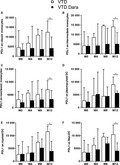

## 1 INTRODUCTION

Targeting CD38 with the monoclonal antibody daratumumab (Dara) is a widely used treatment in the setting of relapsed or refractory multiple myeloma (MM).^1^, ^2^, ^3^, ^4^, ^5^, ^6^ Therefore, Dara is currently approved, in combination with lenalidomide or bortezomib, for treatment of patients with MM who have received at least one prior line of treatment. Dara mediates the death of CD38‐expressing MM cells through a variety of immune‐mediated mechanisms (antibody‐dependent cell‐mediated cytotoxicity, complement‐dependent cytotoxicity, and antibody‐dependent cellular phagocytosis) and direct apoptosis by crosslinking.^7^, ^8^, ^9^ Furthermore, several studies have demonstrated that beside a direct antitumor effect, Dara probably have an immunologic mechanism of action through targeting of CD38 expressing non‐MM immune cells.^10^, ^11^

Recently, Chen et al^12^ demonstrated that CD38 upregulation in tumor induces resistance to programmed cell death 1 (PD‐1)/programmed cell death ligand 1 (PD‐L1) blocking antibodies and coinhibition of CD38 and PD‐L1 improves antitumor immune responses. This mechanism of immune resistance caused by CD38 provides an evident rationale to evaluate immunomodulatory effects of CD38 blockade by Dara on the PD‐L1 expressing immune cells. Several studies have demonstrated that MM cell (either cell lines or primary cells) upregulate PD‐L1, and its receptor PD‐1 is found on a proportion of T cells.^13^, ^14^, ^15^, ^16^ Moreover, dendritic cells (DC) in MM bone marrow expressed PD‐L1 and induced a suppression of PD‐1‐expressing T‐cell and NK‐cell immune functions by engaging immune checkpoints via the PD‐L1/PD‐1 signaling axis.^17^ Herein, we aimed to assess the immunomodulatory effects of Dara on CD38 expressing immune cells in the blood of newly diagnosed MM patients receiving Dara based combination therapy.

## 2 MATERIALS AND METHODS

### 2.1 Patients and healthy donors

Between June 2016 and July 2017, 18 consecutive patients with newly diagnosed untreated MM were analyzed in this single‐center study. In the bortezomib‐thalidomide‐dexamethasone (VTD) group, all patients received 4 cycles (1 cycle = 4 weeks) of VTD, autologous stem cell transplant after high dose chemotherapy (melphalan), followed by consolidation with two cycles of VTD, no maintenance was administered. In the VTD‐Dara group, patients received the same treatment with the addition of intravenous Dara (16 mg/kg) weekly during the first two cycles and then every 2 weeks. Written informed consent was obtained from each patient. The study was approved by the local institutional review board. A group of eight healthy donors (HD group, Etablissement Français du Sang (EFS), Paris Saint‐Antoine‐Crozatier) was established to compare CD38 expression with MM patients.

### 2.2 Biological samples

Peripheral blood samples were collected in EDTA tubes (BD Biosciences) at baseline (immediately before the first administration of treatment) and at 4, 8, and 12 weeks of treatment. Peripheral blood mononuclear cells (PBMCs) were isolated with a standard gradient centrifugation procedure on a lymphocyte separation medium (Lymphosep separation media, Dutscher), and an aliquot was cryopreserved for storage.

### 2.3 Phenotype analysis by flow cytometry

**Figure 1:**
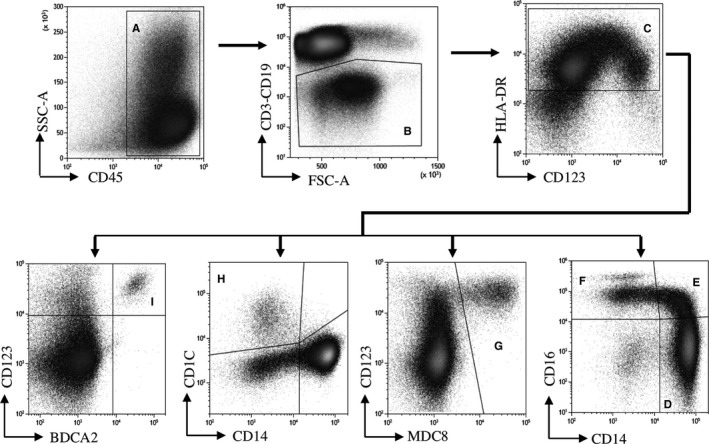
Gating strategy for analysis of dendritic cell and monocyte subpopulations. Gating was sequentially done on CD45^+^ (A) and negativity for CD3^+^ CD19^+^ lineage (B). HLA‐DR^+^ cells were selected (C), and monocyte subtypes were discriminated according to the expression of CD16 and CD14 in HLA‐DR^+^ cells. Classical monocytes are CD16^−^ CD14^+^ (D), intermediate monocytes CD16^+^ CD14^+^ (E), and nonclassical monocytes CD16^+^ CD14^−^ (F). Slan‐DC were identified in HLA‐DR^+^ cells as M‐DC8^+^ (G), myeloid DC as CD1c^+^ (H), and plasmacytoid DC as CD123^+^ BDCA2^+^ (I)

**Figure 2:**
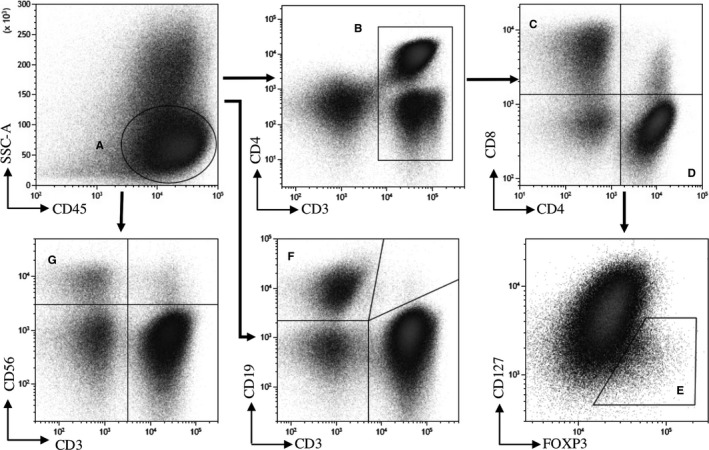
Gating strategy for analysis of lymphocyte subpopulations. Gating of lymphocytes was sequentially done on CD45^+^ and low SSC‐A (A). CD3^+^ were selected (B), and CD4+ (D) and CD8^+^ (C) cells were discriminated according to the expression of CD4 and CD8 in CD3^+^ cells. Tregs cells were identified in CD4^+^ cells as CD127^−^ CD25^+^ FOXP3^+^ (E). Finally, B cells and natural killer cells were selected on CD3^−^ cells for their positivity for CD19 (F) and CD56 (G), respectively

The phenotype of T, B, and NK cells, monocytes and DCs was determined after thawing of PBMC using the following fluorochrome‐conjugated antibody panels: for myeloid cells analysis, CD1c FITC (Ozyme, Biolegend); MDC8 PE and BDCA2 PC7 (Miltenyi); CD123 ECD, CD38 PC5.5, CD56 AA700, CD3^+^ CD19 AA750, and CD14 PB (Beckman Coulter); CD274 (PDL1) APC, CD45 BV510, HLA‐DR BV605, CD86 BV650, and CD16 BV786 (BD Biosciences). For lymphoid cells analysis, CD127 FITC and CCR7 VioBlue (Miltenyi); CD45RA ECD, CD38 PC5.5, CD56 AA700, CD3 AA750, and CD8 KRO (Beckman Coulter); CD25 PC7, CD45 BV605, CD4 BV650, and CD19 BV786 (BD Biosciences). For Treg analysis, cells were permeabilized and incubated with FOXP3 PE (Beckman Coulter) and Ki67 A647 (BD Biosciences). In addition, PD‐L1 expression was evaluated on each cell subset using median fluorescence intensity (MFI). Data were acquired on a CytoFLEX flow cytometer (Beckman Coulter) and analyzed using Kaluza Analysis v2.1 software (Beckman Coulter). To calculate absolute values of cells per microliter of blood, the numbers of lymphocytes on routine clinical blood counts from the same day were collected from the patient charts. Based on these parameters, we estimated what proportion of cells in the blood of the patients was represented by each cell type analyzed by flow cytometry. Gating strategies used to identify myeloid and lymphoid immune subpopulations are described in Figures 1 and 2. The data that support the findings of this study are available from the corresponding author upon reasonable request.

### 2.4 Statistical analysis

Data are expressed as median and interquartile range confidence intervals, and numbers with their frequencies. All comparisons were made using the nonparametric Mann‐Whitney *U* test for unpaired data. All statistical analyses were performed using GraphPad Prism 8.2 (Graphpad Software). A *P* < .05 was considered as statistically significant.

## 3 RESULTS

### 3.1 Patients

Patients characteristics are described in Table 1. Nine consecutive MM patients were included in each group. Median age of patients was 56 (range, 37‐66) years in the VTD‐Dara group versus 66 (range, 50‐67) years in the VTD group (*P* = .01). The two groups were comparable regarding gender and cytogenetic risk. The median follow‐up among surviving patients was 20 (range: 7‐30) months. All patients achieved at least partial response, and only one patient in the VTD group relapsed at five months. This patient presented a specific pericarditis and cutaneous plasmacytomas associated with a refractory MM and finally deceased despite various combinations of proteasome inhibitor, immunomodulatory drugs, and Dara. No other death was reported in this cohort of patients.

**Table 1:** Characteristics of patients

Group	Patient ID	Gender	Age	Isotype	Cytogenetic	ISS
VTD	1	Male	67	IgG Kappa	Normal	2
VTD	2	Female	66	IgG Lambda	Normal	3
VTD	3	Male	62	IgG Kappa	Normal	3
VTD	4	Female	61	IgG Kappa	Normal	1
VTD	5	Male	70	IgA Kappa	Monosomy 13	2
VTD	6	Female	66	IgG Kappa	Normal	1
VTD	7	Female	64	IgA Lambda	Normal	1
VTD	8	Female	67	IgG Lambda	t (11,14)	3
VTD	9	Female	50	IgG Kappa	Normal	1
VTD‐Dara	10	Female	54	IgG Lambda	Normal	1
VTD‐Dara	11	Female	56	IgG Kappa	Normal	1
VTD‐Dara	12	Female	56	IgG Lambda	Normal	1
VTD‐Dara	13	Male	42	IgG Kappa	Normal	1
VTD‐Dara	14	Female	56	IgG Lambda	>3 abnormalities	1
VTD‐Dara	15	Male	37	Lambda light chain	Normal	1
VTD‐Dara	16	Female	62	IgG Kappa	Normal	1
VTD‐Dara	17	Male	66	IgG Kappa	Normal	1
VTD‐Dara	18	Male	57	IgG Lambda	t (4,14), del17p	2

Abbreviations: ISS, international stagingsystem; MM, multiple myeloma; VTD, bortezomib‐thalidomide‐dexamethasone; VTD‐Dara, bortezomib‐thalidomide‐dexamethasone daratumumab.

### 3.2 Dara induces immunomodulatory effects on CD38‐expressing immune cells

**Figure 3:**
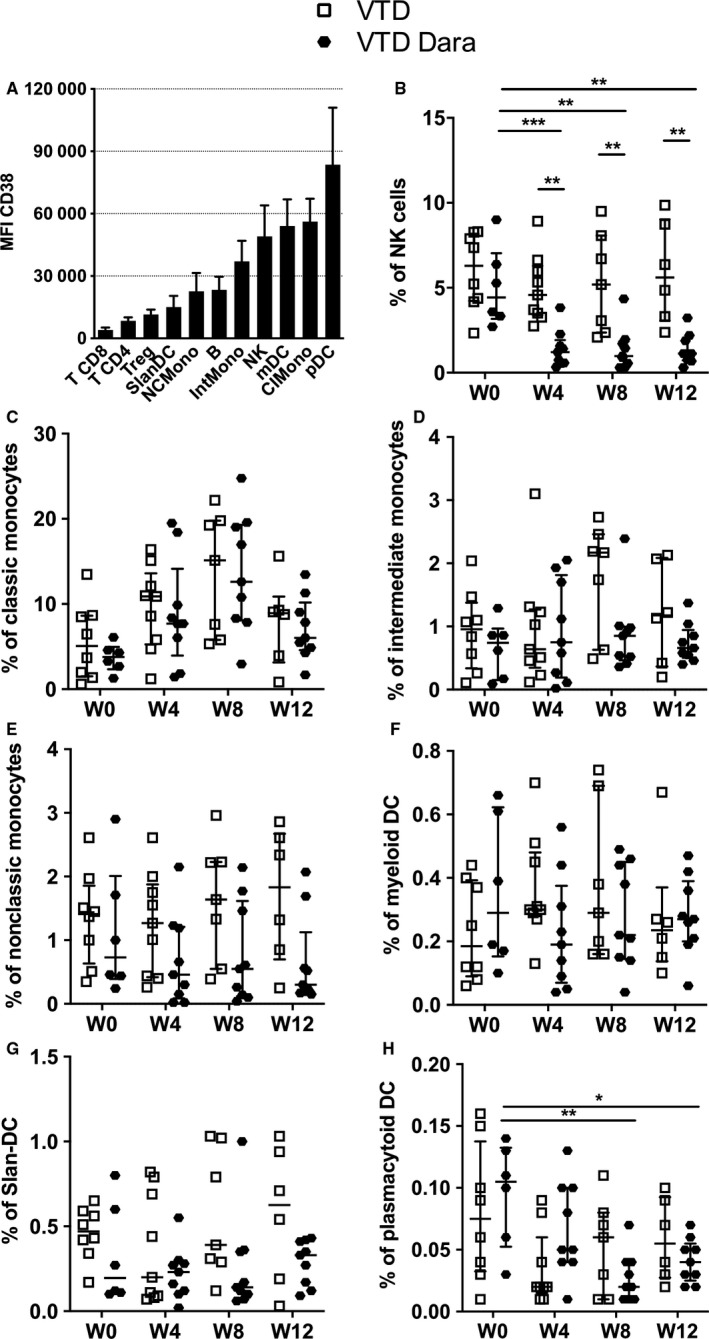
CD38 expression and effects of daratumumab on immune cell populations of multiple myeloma patients. Expression of CD38 in monocytes, dendritic cells, and lymphoid cells in healthy donors and in MM patients (A). Bars display the median CD38 MFI, and interquartile range confidence intervals (error bars) are shown. Proportions of (B) NK cells (CD3‐CD56), (C) classical monocytes (CD14^+^ CD16^−^), (D) intermediate monocytes (CD14^+^ CD16^+^), (E) nonclassical monocytes (CD14^−^ CD16^+^), (F) myeloid dendritic cells (CD1c^+^), (G) Slan‐DC (MDC8^+^), and (H) plasmacytoid dendritic cells (CD123^+^ BDCA2^+^) on MM patients’ lymphocytes or PBMC under combined treatment. The median percentage of PBMC and interquartile range confidence intervals (error bars) are shown. Abbreviations: ClMono, classic monocytes; IntMono, intermediate monocytes; mDC, myeloid dendritic cells; MFI, mean fluorescent intensity; NCMono, nonclassical monocytes; NK, natural killer cells; pDC, plasmacytoid dendritic cells; Slan‐DC, 6‐sulfo LacNac dendritic cells

We first evaluated expression of CD38 on T, B, NK cells, monocytes, and DC in PBMC of newly diagnosed MM patients and healthy donors. We found similar levels of CD38 expression on myeloid and lymphoid immune cells from HD and MM patients (data not shown). Looking at the mean fluorescent intensity of CD38 on these cellular populations, we observed that plasmacytoid dendritic cells (pDC) expressed the highest levels of CD38, followed by subsets of classical monocytes, myeloid dendritic cells (mDC), and NK cells, while Tregs, and CD4^+^ or CD8^+^ T cells expressed the lowest levels of CD38 (Figure 3A).

We then performed a quantitative analysis of monocytes, DC, and lymphocyte subsets at baseline and at 4, 8, and 12 weeks of treatment. As previously reported, we observed a rapid and lasting depletion of NK cells (*P* = .002) after exposure with Dara (Figure 3B). However, Dara exposure had no significant impact on monocytes, mDC, and 6‐sulfo LacNAc‐positive dendritic cells (Slan‐DC) which expressed an intermediate level of CD38 (Figure 3C‐G). Interestingly, in correlation with the strong expression of CD38 by pDC (Figure 3A), we observed a progressive and significant decrease of pDC (*P* = .009) in the VTD‐Dara group (Figure 3H).

### 3.3 Dara prevents PDL1 expression on CD38‐expressing antigen‐presenting cells

**Figure 4:**
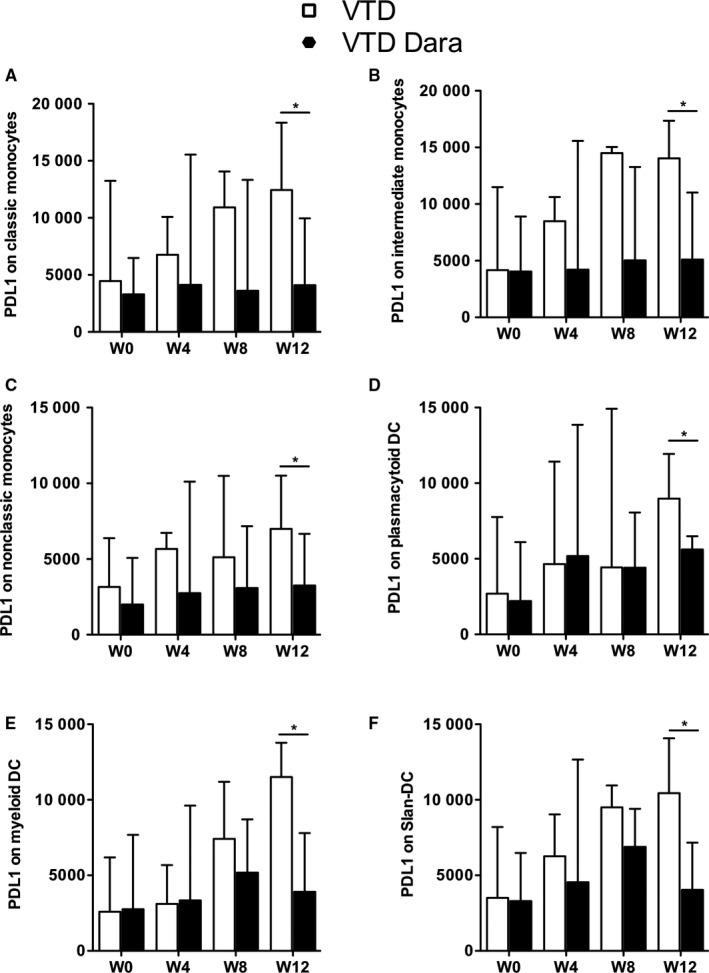
PD‐L1 expression in monocytes and dendritic cells of MM patients. Expression of PD‐L1 in (A) classical monocytes (CD14^+^ CD16^−^), (B) intermediate monocytes (CD14^+^ CD16^+^), (C) nonclassical monocytes (CD14^−^ CD16^+^), (D) plasmacytoid dendritic cells (CD123^+^ BDCA2^+^), (E) myeloid dendritic cells (CD1c^+^), and (F) Slan‐DC (MDC8^+^) in MM patients at diagnosis and after four cycles of treatment. Bars display the median PD‐L1 MFI, and interquartile range confidence intervals (error bars) are shown

To further understand the immunomodulatory activity of Dara, we investigated PD‐L1 expression on the CD38‐expressing antigen‐presenting cells. While we observed a progressive increase of the PD‐L1 expression in monocyte subsets for patients from the VTD group, PD‐L1 expression remained stable in the VTD‐Dara group. After three cycles of treatment, PD‐L1 expression in monocyte subsets was significantly increased in VTD patients compared with patients in the VTD‐Dara group (classical monocytes *P* = .04, intermediate monocytes *P* = .04, and nonclassical monocytes *P* = .03) (Figure 4A‐C). Interestingly, we observed similar findings for DC subsets, with patients in the VTD group exhibiting significantly higher levels of PD‐L1 expression compared with VTD‐Dara patients, after three cycles of treatment (pDC *P* = .03, mDC *P* = .01, and Slan‐DC *P* = .02) (Figure 4D‐F).

## 4 DISCUSSION

CD38 is a membrane molecule with receptor function involved in immune and metabolic functions.^18^ Our results confirm the wide expression of CD38 on immune cells in MM patients and HD.^10^ In addition to the well‐known rapid depletion of NK cells, we found that Dara progressively decreased pDC which strongly expressed CD38. The absence of complete pDC depletion could be attributed to the continuous de novo pDC production. This is particularly interesting given the deleterious effect of pDC in MM. Indeed, it has been shown that pDC confer a growth and survival advantage to MM cells.^19^ More precisely, the pDC antitumor function, through the decrease of cytotoxic CD8 T‐cell activation, is abolished by MM cells.^20^ Therefore, the decreased number of pDC associated with the use of Dara suggests an additional mechanism of action of Dara through pDC depletion. Recently, Chen et al^12^ demonstrated that CD38 upregulation in tumors induces resistance to PD‐1/PD‐L1 blocking antibodies and coinhibition of CD38 and PD‐L1 improves antitumor immune response. Several studies have shown that PD‐L1 is expressed on plasma cells but also on DC in the bone marrow, with a various intensity of PDL‐1 expression among patients.^13^, ^14^, ^15^, ^16^, ^21^ Furthermore, PD‐L1‐expressing pDC are increased and localized with PD‐L1‐positive MM cells in bone marrow.^19^ Importantly, PD‐L1 expression correlates with progression of disease, with the highest levels of expression in relapsed or relapsed/refractory MM.^14^ Overall, this suggests that PD‐L1 positive DC are important in the regulation of tumor‐specific cytotoxic T‐cell responses and contribute to attenuate antitumor immunity in MM patients. In our study, we found that while PD‐L1 expression is increased on peripheral DC and monocytes during VTD treatment, combination with the anti‐CD38 monoclonal antibody Dara seems to prevent this phenomenon. In fact, it has been reported that inflammation can lead to an increased expression of PD‐L1 on DC, suggesting that inflammation induced by chemotherapy could be responsible for the increase PD‐L1 expression observed in the control group upon treatment.^22^ In contrast, in the Dara group, as DC subsets are continuously depleted by Dara, we hypothesized that the low PD‐L1 expression was the reflect of the expression level on de novo produced DC. Given the important expression of Fc receptors (FcR) on DC,^23^ we cannot exclude that the anti‐CD38 monoclonal antibody may react simultaneously on the same cell via their binding site and also via FcR by implementing the so‐called scorpion effect.^24^ Nevertheless, ultimately, we observed a depletion of the DC in our patients upon Dara treatment. Overall, this suggests that targeting CD38 in MM may contribute to restore antitumor immune response mediated by DC and cytotoxic T cells, through a continuous depletion of these cell subsets, preventing PD‐L1 upregulation.

Combination of anti‐CD38 monoclonal antibodies and PD‐1/PD‐L1 blockade to improve disease response was also recently suggested by Verma et al^25^ They reported that PD‐1 blockade in subprimed CD8 cells induced dysfunctional PD‐1^+^CD38^hi^CD8^+^ cells and anti‐PD1 resistance that may be reverted by the use of anti‐CD38 monoclonal antibodies.

Finally, despite a limited number of patients and lack of in vitro mechanistic studies, our results suggest new mechanisms of action of Dara through (a) depletion of pDC and (b) prevention of PD‐L1 expression upregulation on immune cells. Our finding provides new evidences for development of therapeutic strategies targeting both CD38 and PD‐L1/PD‐1 pathway in patients with MM.

## CONFLICT OF INTEREST

The authors declare no competing financial interests.

## **AUTHOR**s’ CONTRIBUTIONS

All authors listed on the manuscript have contributed substantially to this work: NS, BG, MM and FM designed the study, NS collected the data, ZM, MM and FM recruited the patients, NS, BG and LR performed experimental work, NS and FM performed the statistical analysis. NS and FM write the manuscript. All authors analyzed the data, reviewed the manuscript, and agreed to its submission for publication.

## Data Availability

The data that support the findings of this study are available from the corresponding author upon reasonable request.
